# An automated method for the evaluation of breast cancer using infrared thermography

**DOI:** 10.17179/excli2018-1735

**Published:** 2018-10-26

**Authors:** Antony Morales-Cervantes, Eleazar Samuel Kolosovas-Machuca, Edgar Guevara, Mireya Maruris Reducindo, Alix Berenice Bello Hernández, Manuel Ramos García, Francisco Javier González

**Affiliations:** 1 Facultad de Ciencias, Universidad Autónoma de San Luis Potosí, Av. Dr. Salvador Nava Mtz. s/n, Zona Universitaria 78290, San Luis Potosí, SLP, México; 2 Coordinación para la Innovación y la Aplicación de la Ciencia y la Tecnología Universidad, Autónoma de San Luis Potosí, Sierra Leona 550, Lomas 2da. Sección 78210, San Luís Potosí, SLP, México; 3 CONACYT-Universidad Autónoma de San Luis Potosí, Sierra Leona 550, Lomas 2da. Sección 78210, San Luís Potosí, SLP, México; 4 Unidad Académica de Ciencias Naturales, Universidad Autónoma de Guerrero, Carretera Nal. Chilpancingo-Petaquillas, Ex Rancho Shalako 39105. Petaquillas, Guerrero, México; 5 Hospital General "Dr Raymundo Abarca Alarcón" Chilpancingo, Carretera Federal Chilpancingo- Zumpango. Paraje Tierras Prietas S/N, Guerrero, México

**Keywords:** breast cancer, infrared thermography, automated screening

## Abstract

Breast cancer is one of the major causes of death for women. Temperature measurement is advantageous because it is non-invasive, non-destructive, and cost-effective. Temperature measurement through infrared thermography is useful to detect changes in blood perfusion that can occur due to inflammation, angiogenesis, or other pathological causes. In this work, we analyzed 206 thermograms of patients with suspected breast cancer, using a classification method, in which thermal asymmetries were computed, the most vascularized areas of each breast were extracted and compared; then these two metrics were added to yield a thermal score, indicative of thermal anomalies. The classification method based on this thermal score allowed us to obtain the test sensitivity of 100 %, specificity of 68.68 %; a positive predictive value of 11.42 % and negative predictive value of 100 %. These results highlight the potential of thermography imaging as adjunctive tool to mammography in breast cancer screening.

## Introduction

Breast cancer is one of the major causes of death for women (Rastghalam and Pourghassem, 2016[18]), affecting all social levels of the population. Breast cancer is the most common cancer type in Mexico and around the world (INEGI, 2015[13]; WHO, 2018[21]). It has been shown that the probability of a person developing breast cancer depends on factors such as age, gender and genetics; which unfortunately cannot be avoided. There are some typical features of cancer, such as tumour and specific tissue activity that specialists use to perform an early diagnosis (Guzman-Cabrera et al., 2016[11]). The early detection of cancer ensures a better prognosis and a higher survival rate. Indeed, if it is detected in early stages the cure rate is 95 % (Gautherie, 1980[7]).

Breast imaging techniques have been developed as primary clinical methods for the identification of early-stage breast cancers as well as differentiated from benign breast tumours (Yao et al., 2014[23]). Furthermore, mammography is the most common image technique used for breast cancer screening. However, the rate of false negatives can reach values up to 30 %, presenting some disadvantages like expose of patients to ionizing radiation and discomfort (Boquete et al., 2012[2]). In addition, mammography is less effective in younger women and those who have dense breast tissue (Wishart et al., 2010[22]). On the other hand, ultrasound techniques are mainly used to differentiate the properties of cystic and solid mammary lesions identified by mammography. In those studies, dense breast tissue can be examined by using biopsy guided by aspiration and preoperative localization. Therefore, ultrasound alone is not suitable as a screening method for breast cancer because of the amount of time required to perform an examination, the need for qualified personnel in carrying out the procedure, and other limitations. In fact, ultrasound and mammography may overlook many cases in which the tumour is < 0.5 cm (Yao et al., 2014[23]).

In the 1960s infrared thermography began to be used in medical diagnosis, however, it was not until 1982 that it was approved by the Food and Drug Administration (FDA) as a complementary tool for the detection of breast cancer (Arora et al., 2008[1]). Since then, the sensitivity of the infrared imaging technology has increased substantially becoming a more powerful tool for the diagnosis of breast cancer (González, 2011[10]). Though, temperature measurement through infrared thermography is advantageous as it is completely non-invasive, non-destructive, cost-effective and can provide temperature data giving a distribution over a wide surface (Han et al., 2015[12]). The thermal analyses of skin temperature distribution, with the goal of obtaining the information on a possible inner tumour, offers more advantages to indicate abnormal metabolism in early stages of cancer (Chavez et al. 2010[3]). Thus, it could be stated that thermography is a convenient technique to localize changes in blood perfusion that can occur due to inflammation, angiogenesis, or other causes that could generate asymmetries in the temperature distributions. These asymmetries as well as the presence of hot and cold temperature points spots are known to be a good indicator of an underlying problem (Schaefer, 2014[19]).

While mammography and ultrasound diagnoses are typically performed manually by experts, there is a high demand for automated methods to provide an objective solution that could be used as a second opinion (Krawczyk and Schaefer, 2014[16]). Some automated methods rely on the analyses of thermograms by dividing the image into segments of interest and subsequently analysing them.

Hereafter, the segmentation of images refers to the technique that divides a digital image into multiple sections and it is usually used to identify regions of interest as well as relevant features of information in digital images (Zhang et al., 2015[24]). One of the main ways to differentiate abnormalities in the breasts is the comparison by thermal asymmetries where the left breast is compared with the right breast. 

For instance, recent analyses for breast cancer detection have obtained 85 % sensitivity and 61.53 % positive predictive value using thermography, such as fuzzy active contours (Ghayoumi Zadeh et al. 2016[9]) Markov random field analyses have obtained 80 % sensitivity and 85 % specificity (Rastghalam and Pourghassem, 2016[18]). Active contour analyses have been shown to attain 90 % sensitivity and 54 % specificity (Ghayoumi Zadeh et al. 2016[8]). Texture feature analyses have yielded a sensitivity of 83 % and specificity of 83 % (Francis et al., 2014[6]), and studies based on texture feature combined with minimum variance quantization have obtained 79 % sensitivity and 100 % specificity (Milosevic et al., 2014[17]). In this work, we present an automated method for the evaluation of breast cancer using infrared thermography, the breast thermograms are automatically analysed using image processing techniques. Table 1(Tab. 1) (References in Table 1: Ghayoumi Zadeh et al., 2016[9]; Rastghalam and Pourghassem, 2016[18]; Ghayoumi Zadeh et al., 2016[8]; Francis et al. 2014[6]; Milosevic et al., 2014[17]) shows the comparison between this work and the mentioned papers.

## Materials and Methods

The participants in the present study had clinical evidence of a tumour suggestive of cancer, risk factors for breast cancer and they were heading for consultation. None of the patients declared cancer at the time of inviting them to participate in the study (General Hospital "Dr. Raymundo Abarca Alarcón" Chilpancingo, Guerrero, Mexico). The study was approved by the hospital ethics committee. Furthermore, the patients read the informed consent form before they signed it. It is worthy to mention that patients did not perform any physical activities, ingest alcohol, smoke or use deodorant one day before thermal imaging was carried out. Indeed, an acclimation process was performed prior to the thermal images acquisition, during the imaging process the patients were asked to remain uncovered from the waist upwards for 20 minutes in a room with a controlled temperature of 24±1 °C. It was intended to prevent direct airflow to the patient and avoid noise from instruments that emitted heat. During the thermographic imaging, the patients were instructed to stand, and keep the hands holding their neck, 1.5m away from the camera. As a total of 5 images were taken, including; one frontal, one left lateral, one right lateral, and two breasts separately (frontal). The characteristics of overall patient population are summarized in Table 2(Tab. 2).

Once the thermographic images were taken, a previously scheduled mammogram was performed at the same location. Following the procedure, a radiologist performed the BI-RADS classification of the mammography and clinical diagnosis as well as biopsy were performed to the patients with a suspicious abnormality in the evaluation. 

The camera used for imaging was the IR FlexCam Pro® with a Focal Plane Array (FPA) detector, based on Vanadium Oxide (VOX) Uncooled Microbolometer, thermal sensitivity @ 30Hz: = 0.070 °C at 30 °C, Temperature Range -20 °C to 100 °C, ± 2 % accuracy and a lens of 20 mm f / 0.8 Germanium with a Field of view of 23 ° Horizontal x 17 ° Vertical. The emissivity was set to 0.97 (Jones, 1998[14]).

The automated program was developed on a MATLAB (The MathWorks, Natick, MA) platform. It is based on the interpretation of thermal images by using a thermal score derivative from the scale of the infrared classification Ville Marie (Keyserlingk et al. 1998[15]; González, 2011[10]). This thermal score considers the two most significant infrared data: (a) the difference in surface temperature point with higher temperature as compared to the mirror image site in the contralateral breast (ΔT), and (b) the vascular pattern in both locations around and at the site that point (Wang et al., 2010[20]).

Afterward, the thermal score is calculated by adding the amount of vascularization to the difference in surface temperature (degrees Celsius) at the site of the lesion compared to that of the contralateral breast. During the procedure, the amount of vascularization is determined using the following scale:

1. Absence of vascular patterns.
2. When symmetrical or moderate vascular patterns were found.
3. In the case of significant vascular asymmetry.
4. Vascular asymmetry extended in at least one third of the breast area.

The method used for segmentation of the left and right breast imaging and of the rest of the body is based on the analyses of the projection profile using the Sobel operator (Dayakshini et al., 2015[5]). It is useful to find the upper, lower, left and right edges of the detected border of the breast thermographic image.

### Edge detection with operator sobel

**Figure 1:**
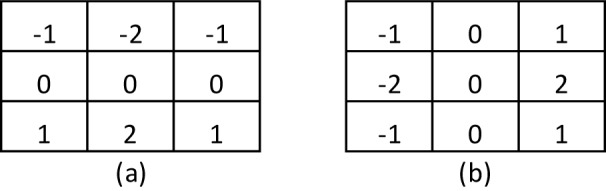
Sobel Masks. a) Horizontal edge and b) Vertical edge detection

To further understand the method, the Sobel operator performs a measurement of the 2-D spatial gradient of the thermal image and emphasizes the regions of high spatial frequency that correspond to the edges of the images itself. Here, the operator consists of a pair of 3x3 masks to make the convolution as shown in Figure 1(Fig. 1). Two masks are applied in the process, the second one rotated by 90 ° with respect to the former one. These masks are designed to respond maximally to edges that run vertically and horizontally with respect to the pixel grid.

It has been shown that the Sobel operator gives smoothing effect (average filter) and reduces false edges. In fact, the image gradient f (x, y) is a vector and is given by,


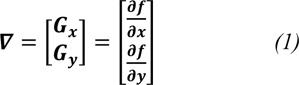


where ∇ is the gradient operator.

Here, the magnitude of the gradient is given by,


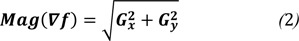


where Mag (∇f) gives the magnitude of the edge to a particular location x-y.

The direction of the edge is found by,


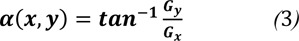


**Figure 2:**
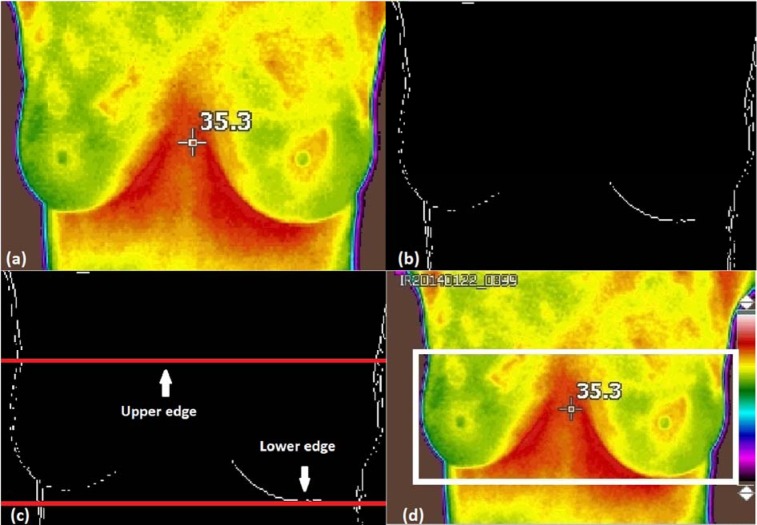
Figure 2: a) Original image, b) Binary image with detected edges, c) Upper and lower limits detected and d) upper, lower, left and right edges of the detected border are shown on the highlighted area.

Figure 2(Fig. 2) shows the results of edge detection of the breast thermogram image. The boundary of the breast thermogram image is successfully detected using the Sobel operator as shown in Figure 2b(Fig. 2).

### Detection of the lower part of the breasts

To begin with the segmentation of the breasts, we converted the image into a grayscale to be able to apply Sobel and detect the edges. Secondly, to locate the top and bottom edges of the image we used the horizontal projection profile (HPP). Meanwhile, the Vertical Projection Profile (VPP) was used to find the left and right edges. The procedure is described as follows:

To find the lower part of the breast, the edges detected in the image are scanned horizontally, to count the number of white pixels in each row of the lower part of the image. Then, in the infra-mammary line, the number of white pixels increases due to the infra-mammary fold of the breast. Later, the scan is repeated until the HPP value has been equal or greater than the preset value is obtained, chosen by tests when observing that it performs the detection of the lower edge of the breasts, the row number corresponding to the first high HPP value is taken as the lower limit (LL) for the segmentation of the breast thermographic image. Therefore, the HPP value will be less below the infra-mammary border of the breast.

### Axillary detection (upper edge detection) of the breast

Normalization of breast height is necessary due to the variable height of the images. To standardize the height in the image, the distance between the lower edge detected and the bottom of the image is measured. The value of the distance varies depending on the structure and size of the breast. The HPP is used again to find the upper border of the breast. For instance, Dayakshini and colleagues (2015[5]) have shown that the distance value will be high for small breasts and less for large breasts, according to the study and observation, the height of the breast is calculated as indicated below (Dayakshini et al., 2015[5]).

If the distance between the bottom of the image and the lower limit of the breast is less than 26 % of the total pixels height, then


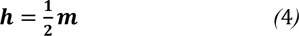


where *h* is the height of the image and *m* is the total number of rows present in the image.

If the distance between the lower part of the image and the lower limit of the breast is greater than 26 % of the total pixels height, then


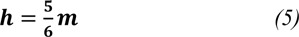


The upper limit (UL) will be located in the row position given by,


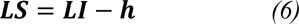


Finally, the upper and lower limits detected are shown in Figure 2c(Fig. 2). Horizontal lines along the upper and lower limits are represented on the images and do not represent actual data.

### Detection of right and left edges

Now, the breast thermographic image is segmented from the unwanted upper and lower part, by using the upper and lower limits. The left and right edges are detected from the image using the vertical projection profile (VPP) method. This algorithm (VPP) is defined as the white pixel count number for each column. The steps followed for the detection of the left edge are given below.

1. Segmentation of the image with the top and bottom edges and apply Sobel.

2. To find the left limit, the image is analysed from right to left and if a white pixel is found, the position of the last column where it was found is stored, pointing only to the white pixel found further to the left.

3. To find the right limit, the image is analysed from left to right and if a white pixel is found, the position of the last column where it was found is stored, pointing only to the white pixel found further to the right. Thus, the left and right edges detected are used to form a thermographic image of the desired breast by removing the left and right unwanted part. Moreover, the central axis of the breast is determined by dividing the width of the new image divided by two. Finally, a thermographic image of the breast with the highlighted edges lower, upper, left and right are shown in Figure 2d(Fig. 2).

### Calculation of thermal vascularity

**Figure 3:**
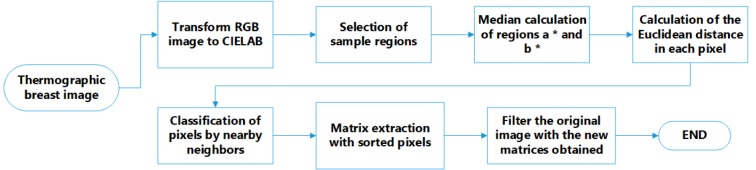
Process diagram to segment the vascularity of the sinus in the CIE L * a * b * space

An important part of imaging processing consists of the calculation of thermal vascularity. This process is performed in the colour space known as CIELAB which is normally used to describe all the colours that the human eye can perceive. Indeed, it is performed by removing the reddest parts of the image. The block diagram of this process is shown in Figure 3(Fig. 3).

Figure 2d(Fig. 2) shows the areas with the highest temperature. The colour space L * a * b * (also known as CIELAB or CIE L * a * b *) allows the quantification of visual differences. In this colour model, the space is defined by three variables, L *, representing the brightness, and a * and b * that correspond to the components of the tonality. The value of a * defines the distance along the red-green axis, while the value of b * represents the distance along the blue-yellow axis, these axes specified what is called the CIEXYZ space (Cuevas et al., 2010[4]). 

**Figure 4:**
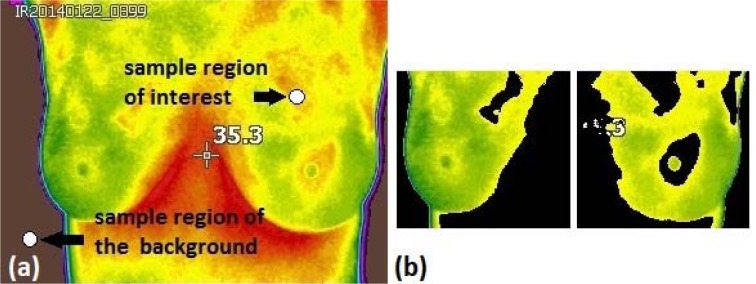
a) The thermal image shows the sample region used to find the vascularity and to eliminate the background in the images and b) Segmented breasts with eliminated vascularized area.

During the calculation of thermal vascularity, first, the image of the RGB space is converted into L * a * b * space. Then, the sample regions of interest are selected to obtain the a * b * values of the selected colours as shown in Figure 4a(Fig. 4). Later, the mean values of a * and b * of the area of the selected samples is calculated, these values serve as markers for space a * and b *. Next, each pixel is classified by calculating the Euclidean distance between that pixel and the marker. For instance, if the distance is very small, then the pixel will be labelled as red or background. Thus, an array is created containing the pixels selected by the classification, which are used to filter the original image.

Subsequently, the masks created in the background and red regions are used to segment the image by colour as well as to find the vascularity. Once the original image is segmented by colours, the same values (upper, lower, right, left, and center) are used to separate the breasts contours and vascularized areas aiming to calculate the thermal score (Figure 4b(Fig. 4)).

**Figure 5:**
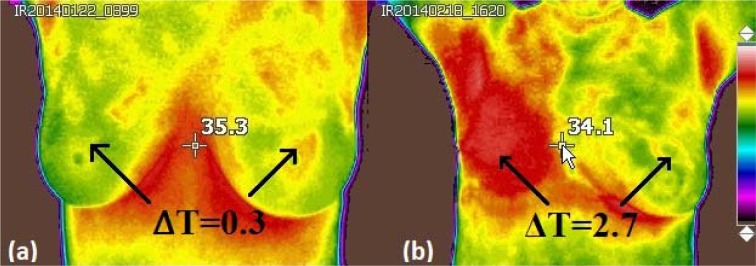
a) IR image of a healthy breasts. (ΔT) represents the difference in the surface on the breast. A calculated thermal score was found to be 2.3, obtained by adding the amount of vascularity (2): The two value denotes symmetrical or moderate vascular patterns, meanwhile a 0.3 value correspond to the difference in surface temperature, ∆T, at the lesion site compared to the contralateral breast and b) IR image of patient with infiltrating ductal carcinoma. The calculated thermal score was assigned to be 6.7, obtained by adding the amount of vascularity (4): vascular asymmetry extended in at least one third of the breast area and a value of 2.7, associated with the difference in temperature surface, ∆T, at the lesion site compared to the contralateral breast.

Finally, the temperature difference of the thermal focus located in one of the breasts with respect to its contralateral part is measured. This is done with the thermal camera at real time, *i.e*. taking the images, observing it in one breast and then in its counterpart to obtain the temperature difference of interest. Then, a delta temperature is added to the thermal score (with a value of 2) of the previously found vascularization as shown in Figure 5a(Fig. 5).

## Results and Discussion

All patients with a BI-RADS suspicious of cancer, underwent biopsy. From those patients, 8 of them exhibited infiltrating ductal carcinoma. Thermograms were analysed using the thermal score as presented in the previous section. Here, the infrared images were divided into two groups, (1) those with a thermal score lower than 2.5 were classified as healthy, (2) those with a thermal score greater than or equal to 2.5 were classified with some anomaly. Once the classification is performed in all the images, a final score is assigned to the corresponding thermal image, for instance, Figure 5b(Fig. 5) shows a cancer patient with a thermal score of 6.7.

Further analysis of the thermographic images reveals statistical data as follows: From 206 patients, 8 true positives and 62 false positives were found. Moreover, 136 were classified as true negatives and there were not any false negatives. Obtaining a sensitivity of 100 % with a specificity of 68.68 %, a positive predictive value of 11.42 % and a negative predictive value of 100 %. 

**Figure 6:**
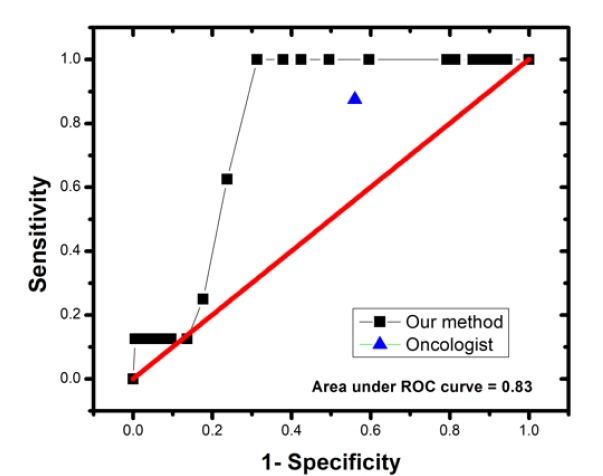
ROC curve of the automated program for classification of thermograms

Figure 6(Fig. 6) shows the receiver-operating characteristic (ROC) curve of the thermograms analyses using the automated program. Based on the results the ROC curve allows us to infer that it is possible to increase the specificity with this method by moving the cut point of the test, otherwise, the sensitivity would decrease considerably. 

It is worthy to mention that the same thermographic images were analysed qualitatively by an oncologist in a double-blind study, his findings were 7 true positives and 87 false positives, while 111 were classified as true negatives and 1 false negative. The comparison of results of the qualitative and quantitative method with our method is shown in Table 3(Tab. 3).

## Conclusions

A classification of thermographic images for breast cancer detection was performed by an automated program. 206 patients were considered for test screening with clinical evidence of a tumour risk factors for breast cancer, BI-RADS classification of mammography, clinical diagnosis and pathology results of the biopsy. The analysed thermograms were classified as healthy (< 2.5 thermal score) or with an abnormality (≥ 2.5 thermal score). The findings revealed that patients who were classified as healthy, positively indicated a healthy state (automated program) with a sensitivity of 100 % and a specificity of 68.68 %. In contrast, the same images analysed qualitatively by an expert, showed a sensitivity around 87.5 % and specificity about 56 %, thus, our results showed a significant improvement over a manual procedure. 

Furthermore, an automated method to analyse thermograms was implemented, increasing the sensitivity and specificity of the test under study. The main goal of it will be to help experts by assisting them with a better screening tool or even gives the possibility that someone without experience may benefit from the test results. The authors emphasize that infrared thermography is not intended to replace mammography, but it is an excellent primary method/technique prior to subjecting patients to x-rays. It could be envisioned as a complementary diagnostic method to improve breast cancer detection.

## Acknowledgements

This work was supported in part (A M-C) by a doctoral grant from CONACYT, by Cátedras CONACYT project 528 (E.G.) and by the Terahertz Science and Technology National Lab (LANCYTT).

## Conflict of interest

The authors declare that there is no conflict of interest.

## Figures and Tables

**Table 1:**
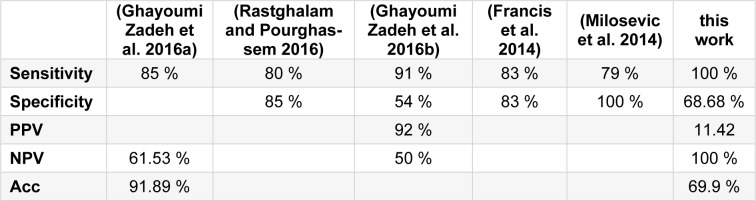
Comparison of this work with recent studies

**Table 2:**
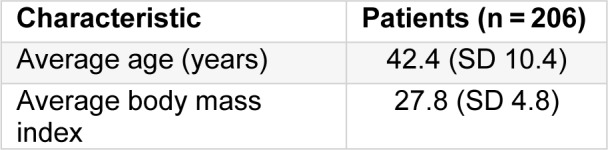
Baseline characteristics of population of a breast cancer study

**Table 3:**
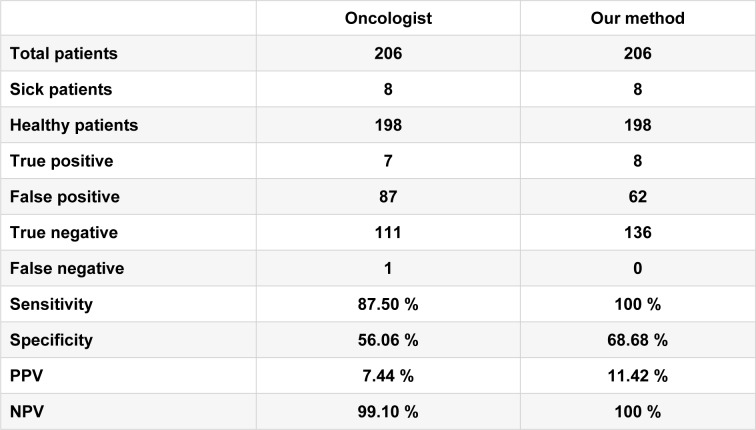
Comparison between our automated method and qualitative evaluation by an oncologist
